# Numerical Investigation of CsSnI_3_ Absorber Layer Parameters Toward Efficient Tin-Based Perovskite Solar Cells

**DOI:** 10.3390/molecules31183204

**Published:** 2026-09-11

**Authors:** Zhongjie Wang, Zihan Tao, Changxin Sun, Hengyu Liu, Xinru Wang, Xinrui Guan, Songyang Ma, Benxiong Hu, Yunxiang Zhang, Qinfang Zhang

**Affiliations:** 1 School of Materials Science and Engineering, Yancheng Institute of Technology, Yancheng 224051, China; wangzj@ycit.edu.cn (Z.W.);; 2 School of Art and Meida, Yancheng Polytechnic College, Yancheng 224005, China; 3 School of Art, Southeast University, Nanjing 211189, China

**Keywords:** perovskite solar cells, CsSnI_3_, TiO_2_ ETL, defect density, P3HT

## Abstract

Lead-free tin-based perovskites, particularly CsSnI_3_, offer a promising route toward environmentally benign photovoltaics, yet their device performance still lags behind lead-based counterparts. In this work, we perform a systematic SCAPS-1D simulation study on a planar heterojunction CsSnI_3_ solar cell with a TiO_2_ electron transport layer and a P3HT hole transport layer. We focus on the interplay between absorber-layer properties, acceptor doping concentration, thickness, defect density, carrier mobility, and parasitic resistances. Our results reveal that an optimal acceptor density around 10^19^ cm^−3^ balances built-in potential enhancement against Shockley–Read–Hall recombination, yielding the highest efficiency. Thicker absorbers improve light harvesting but aggravate bulk recombination, while defect densities above 10^16^ cm^−3^ cause catastrophic performance collapse. High carrier mobility (>1 cm^2^/V·s) is essential for efficient collection, and series resistance must be kept below 2 Ω·cm^2^ to avoid fill factor degradation. Under optimized conditions, the device achieves a theoretical efficiency exceeding 27%, demonstrating the critical role of co-optimizing absorber parameters for high-performance, lead-free perovskite solar cells.

## 1. Introduction

As global fossil energy becomes increasingly scarce, and with the serious environmental problems such as greenhouse effect and air pollution caused by the combustion of fossil fuels, the development of clean and renewable energy has become an urgent need for the sustainable development of human society [1,2,3]. Among various alternative energy sources, solar energy, due to its abundant resources, wide distribution, and flexible utilization methods, is regarded as one of the most promising choices [4,5]. The utilization methods of solar energy mainly include photocatalysis (converting into chemical energy) [6,7,8], photovoltaic cells (directly converting into electrical energy) [9,10,11], and photothermal conversion (converting into thermal energy) [12,13,14,15]. Among them, photovoltaic cells have become the focus of attention in both academic and industrial circles because they can achieve efficient, quiet, and zero-emission power generation. Further, among various photovoltaic technologies, thin-film solar cells are favored due to their low material consumption, simple preparation process, and flexibility [16,17,18,19]. In recent years, organic-inorganic hybrid perovskite materials (such as CH_3_NH_3_PbI_3_) have rapidly emerged as a research hotspot in the field of photovoltaics due to their suitable and tunable direct band gap, high light absorption coefficient, long carrier diffusion length, and good solution processing properties [20,21,22,23,24]. The laboratory-certified photoelectric conversion efficiency of this type of material has risen from the initial 3.8% to over 25% at present, demonstrating a huge commercialization prospect [25,26].

However, most of the currently high-efficiency perovskite solar cells (PSCs) are based on lead (Pb)-containing materials [27]. The toxicity of lead not only poses a potential threat to the environment and human health, but also severely restricts the large-scale application and market access of such cells [28]. Therefore, developing environmentally friendly, low-toxicity, or even non-toxic perovskite photovoltaic materials has become one of the key scientific issues in this field [29]. Among the numerous lead-free candidate materials, the all-inorganic tin-based perovskite CsSnI_3_ stands out due to its excellent photoelectric properties: it has a direct bandgap comparable to that of lead-based perovskites (about 1.3 eV), an extremely high light absorption coefficient (>10^4^ cm^−1^), and a relatively high carrier mobility [30,31]. Compared with the conventional inorganic lead-based perovskite CsPbI3, CsSnI_3_ exhibits a narrower optical bandgap (~1.27–1.30 eV for CsSnI_3_ versus ~1.73 eV for CsPbI_3_) [30,31,32], which is closer to the ideal Shockley–Queisser limit and thus beneficial for solar energy harvesting. In terms of lattice structure, both materials crystallize in the perovskite structure with similar cubic lattice constants (CsPbI_3_: ~6.20–6.29 Å; CsSnI_3_: ~6.21–6.27 Å) [30,31], indicating that they share comparable crystal compatibility and could potentially be integrated into tandem or alloyed device configurations. Therefore, in-depth research on CsSnI_3_-based PSCs is of great theoretical significance and practical value for the development of efficient, stable, and environmentally friendly new photovoltaic technologies. Moreover, the good thermal stability and chemical stability of CsSnI_3_ also make it a unique advantage in tandem cells and flexible devices [33]. Therefore, in-depth research on CsSnI_3_-based PSCs is of great theoretical significance and practical value for the development of efficient, stable, and environmentally friendly new photovoltaic technologies.

In recent years, scholars both at home and abroad have conducted a large number of fruitful experimental and theoretical studies on CsSnI_3_ solar cells [30,33,34,35,36]. For thin-film fabrication, CsSnI_3_ is primarily prepared by solution-based methods such as spin-coating, along with thermal evaporation and chemical vapor deposition (CVD) [30,36]. These techniques are already well-established in the PV industry, making CsSnI_3_ suitable for future manufacturing. However, key challenges remain, including phase stability and Sn^2+^ oxidation, which have been the focus of recent experimental efforts [30,33]. In terms of experiments, researchers have attempted to address the issue of Sn^2+^ easily oxidizing to Sn^4+^ through methods such as component engineering, solvent engineering, additive strategies, and interface modification, and have optimized the film morphology, enabling the efficiency of CsSnI_3_ solar cells to exceed 10% [32]. However, compared with lead-based devices, its performance still has a significant gap, and the repeatability and stability need to be improved. In terms of theoretical simulation, some studies have used device simulation software such as SCAPS and wxAMPS to preliminarily explore the effects of parameters such as the thickness of the CsSnI_3_ absorption layer, defect density, and doping concentration on the performance of the cells [37,38]. However, so far, systematic research on carrier transport mechanisms, band matching of the transport layer, and collaborative optimization of multi-layer structures is still relatively scarce, especially lacking in in-depth quantitative analysis of how the carrier concentration of the CsSnI_3_ absorption layer specifically affects the internal electric field distribution, recombination rate, and carrier collection efficiency of the device.

Therefore, this work employs the one-dimensional solar cell simulation software SCAPS-1D (Solar Cell Capacitance Simulator), version 3.3.05, which is based on the Poisson equation, continuity equation, and drift-diffusion theory, to systematically study the photoelectric performance of CsSnI_3_ planar heterojunction solar cells. Firstly, the key influence of the carrier concentration in the CsSnI_3_ absorber layer on the device performance is focused on, and the carrier generation, transport, and recombination mechanisms under corresponding conditions are deeply analyzed. On this basis, the regulatory laws of the key parameters of perovskite absorber layer (such as thickness, defect density, etc.) on the open-circuit voltage (*V*_OC_), short-circuit current density (*J*_SC_), fill factor (*FF*), and photoelectric conversion efficiency (*PCE*) of the device are further systematically investigated. Through the above research, the physical mechanisms of carrier transport and recombination in CsSnI_3_ solar cells are revealed, the optimization directions of each functional layer are clarified, and theoretical guidance and data support are provided for the experimental design and preparation of efficient and stable lead-free perovskite solar cells.

## 2. Modeling and Simulation

### 2.1. Device Modeling

**Figure 1:**
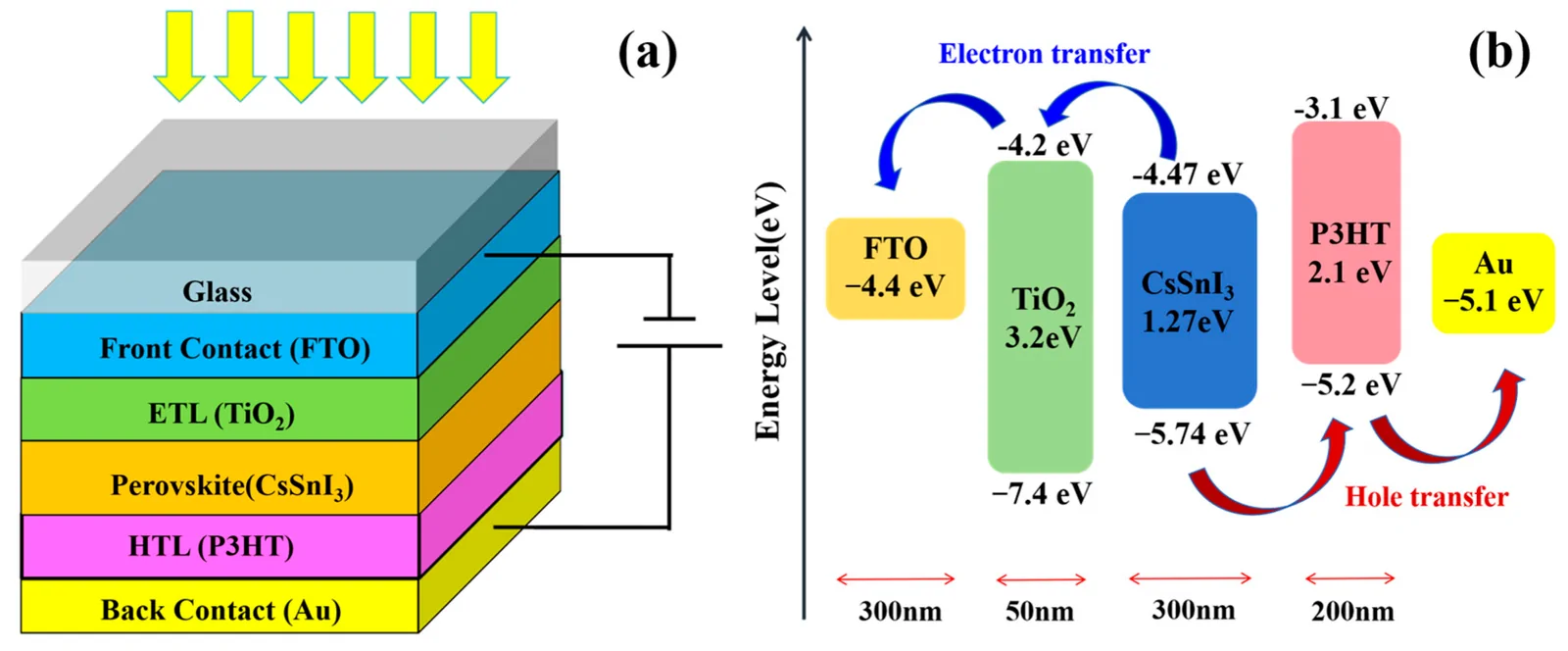
(**a**) Device architecture of the proposed PSCs. (**b**) Charge transfer in solar cells under light irradiation. The schematic is not drawn to scale; the actual layer thicknesses used in the simulations are listed in Table 1.

In this simulation work, the solar cell architecture consists of five stacked layers in the configuration FTO/TiO_2_/CsSnI_3_/P3HT/Au, as illustrated in Figure 1. The FTO (fluorine-doped tin oxide) layer serves as the bottom transparent conductive electrode with a work function of approximately 4.4 eV [39], providing a low-resistance contact between the external load and the electron transport layer. The TiO_2_ layer functions as the electron transport layer (ETL), facilitating efficient extraction and transport of photogenerated electrons from the CsSnI_3_ absorber layer. The CsSnI_3_ layer acts as the perovskite light-absorbing layer, where electron-hole pairs are generated upon illumination. The P3HT (poly(3-hexylthiophene-2,5-diyl)) layer serves as the hole transport layer (HTL), effectively extracting and conveying holes toward the top metal electrode. Finally, the top Au electrode collects holes from the P3HT layer with a work function of approximately 5.1 eV [40], and completes the external circuit, enabling the overall charge-collection process. This multilayer configuration ensures effective charge separation and transport, thereby playing a critical role in determining the photovoltaic performance of the CsSnI_3_-based solar cell.

**Table 1:** Simulated parameters of PSCs.

	FTO [39,41]	TiO_2_ [39]	CsSnI_3_ [39]	P3HT [42]
Thickness, *t* (nm)	300	50	300	200
Band gap, *E_g_* (eV)	3.5	3.2	1.27	2.1
Electron affinity, χ (eV)	4	4.2	4.47	3.2
Relative permittivity, *ε*_r_	9	10	10.59	3
Effective conduction band density, *N*_C_ (cm^−3^)	2.2 × 10^18^	2 × 10^18^	1.58 × 10^19^	2 × 10^21^
Effective valence band density, *N*_V_ (cm^−3^)	1.8 × 10^19^	2 × 10^18^	1.47 × 10^18^	2 × 10^21^
Electron mobility, *μ*_n_ (cm^2^/Vs)	20	250	4.37	0.0018
Hole mobility, *μ*_p_ (cm^2^/Vs)	10	25	4.37	0.0018
Electron thermal velocity, *V*_e_ (cm/s)	1.0 × 10^7^	1.0 × 10^7^	1.0 × 10^7^	1.0 × 10^7^
Hole thermal velocity, *V*_p_ (cm/s)	1.0 × 10^7^	1.0 × 10^7^	1.0 × 10^7^	1.0 × 10^7^
Density of n-type doping, *N*_D_ (cm^−3^)	1.0 × 10^19^	1.0 × 10^18^	0	0
Density of p-type doping, *N*_A_ (cm^−3^)	0	0	1.0 × 10^16^	1.0 × 10^19^
Defect of defects, *N*_t_ (cm^−3^)	1.0 × 10^15^	1.0 × 10^15^	1.0 × 10^14^	1.0 × 10^15^

The selection of TiO_2_ as the ETL and P3HT as the HTL is based on their favorable band alignment with the CsSnI_3_ absorber. TiO_2_ is widely used as an ETL due to its suitable conduction band minimum (CBM), high electron mobility, chemical stability, and low cost. P3HT is chosen as the HTL because its highest occupied molecular orbital (HOMO) level aligns well with the valence band of CsSnI_3_ for efficient hole extraction, and previous simulation studies have shown that P3HT yields the highest efficiency among common HTLs for CsSnI_3_-based devices. As shown in Figure 1b, the CBM of TiO_2_ (−4.2 eV) is slightly higher than that of CsSnI_3_ (−4.47 eV) [39], facilitating electron injection from the absorber to the ETL, while the valence band maximum (VBM) of P3HT (−5.2 eV) is well aligned with that of CsSnI_3_ (−5.74 eV) for efficient hole extraction. This band alignment ensures efficient charge separation and extraction, minimizing interfacial recombination losses.

Within the SCAPS simulation environment, the evaluation of a semiconductor device’s photovoltaic performance relies on the numerical resolution of three governing equations: Poisson’s equation, the electron continuity equation, and the hole continuity equation. These equations collectively describe the electrostatic potential distribution and charge carrier behavior throughout the device [21,39,43,44,45]:

(1) $$\frac{\partial^{2}\varphi }{\partial x^{2}\;}\;=-\frac{\partial E}{\partial x}\;=\;-\frac{\rho }{\varepsilon_{s}}\;=\;-\frac{q}{\varepsilon_{s}}[\left.\;p\;-\;n\;+\;N_{D}\left(x\right)\;-\;N_{A}\left(x\right)\pm N_{\mathrm{def}}\;\left(x\right)\right.]$$

(2) $$\frac{\partial n(x)}{\partial t}\;=G_{n}\left(x\right)-R_{n}\left(x\right)+\frac{1}{q}\frac{\partial J_{n}}{\partial x}$$

(3) $$\frac{\partial p(x)}{\partial t}=G_{p}\left(x\right)-R_{p}\left(x\right)-\frac{1}{q}\frac{\partial J_{p}}{\partial x}$$

The notation employed in the above equations is as follows: *φ* and E stand for the electrostatic potential and the electric field, respectively; *ε*_s_ is the permittivity of the semiconductor material; *p* and *n* denote the hole and electron concentrations; *N*_A_ and *N*_D_ are the doping densities of acceptors and donors; *N*_def_ represents the defect density; *q* is the elementary charge; *J*_n_ and *J*_p_ are the corresponding current densities; *G*_n_, *R*_n_, *G*_p_, and *R*_p_ refer to the generation and recombination rates for each carrier type. Through the self-consistent solution of these equations, key performance indicators of the solar cell are obtained.

### 2.2. Device Simulation

The baseline simulation uses the indicated PSCs structure. Table 1 gives the input parameters for this initial model, taken from theoretical foundations and earlier works. The parameters *V*_e_ and *V*_p_ in Table 1 denote the electron and hole thermal velocities, respectively, which represent the average thermal motion speed of carriers and are used in SCAPS-1D for Shockley–Read–Hall (SRH) recombination calculations. The baseline carrier mobility values in Table 1 are adopted from the literature, while their systematic optimization is discussed in Section 3. The key parameters of the CsSnI_3_ absorber layer, including its band gap of ~1.3 eV and hole mobility of 4.47 cm^2^/Vs, are consistent with experimentally reported values for CsSnI_3_ polycrystalline thin films [30,31,32]. The acceptor doping concentration of 1.0 × 10^16^ cm^−3^ is within the range of Hall effect measurements (~10^17^ cm^−3^) [30], and the defect density of 10^14^ cm^−3^ is a typical assumption for high-quality perovskite films in SCAPS-1D simulations [39,44].

SCAPS-1D is a solar cell simulation program that numerically solves the fundamental semiconductor equations—Poisson‘s equation, the electron continuity equation, and the hole continuity equation under steady-state conditions [39,40]. These coupled differential equations are discretized and solved iteratively using the Newton–Raphson method with appropriate boundary conditions at the contacts. The simulation incorporates key physical mechanisms including Shockley–Read–Hall (SRH) recombination via defect states, radiative recombination, and Auger recombination. The optical generation profile is calculated from the AM1.5G solar spectrum and the wavelength-dependent absorption coefficient of each layer, accounting for reflection and transmission at the multilayer interfaces. The material parameters listed in Table 1 serve as the input variables within this framework, enabling the self-consistent prediction of current density-voltage (J–V) characteristics, external quantum efficiency (EQE), and other photovoltaic performance metrics.

## 3. Result and Discussion

**Figure 2:**
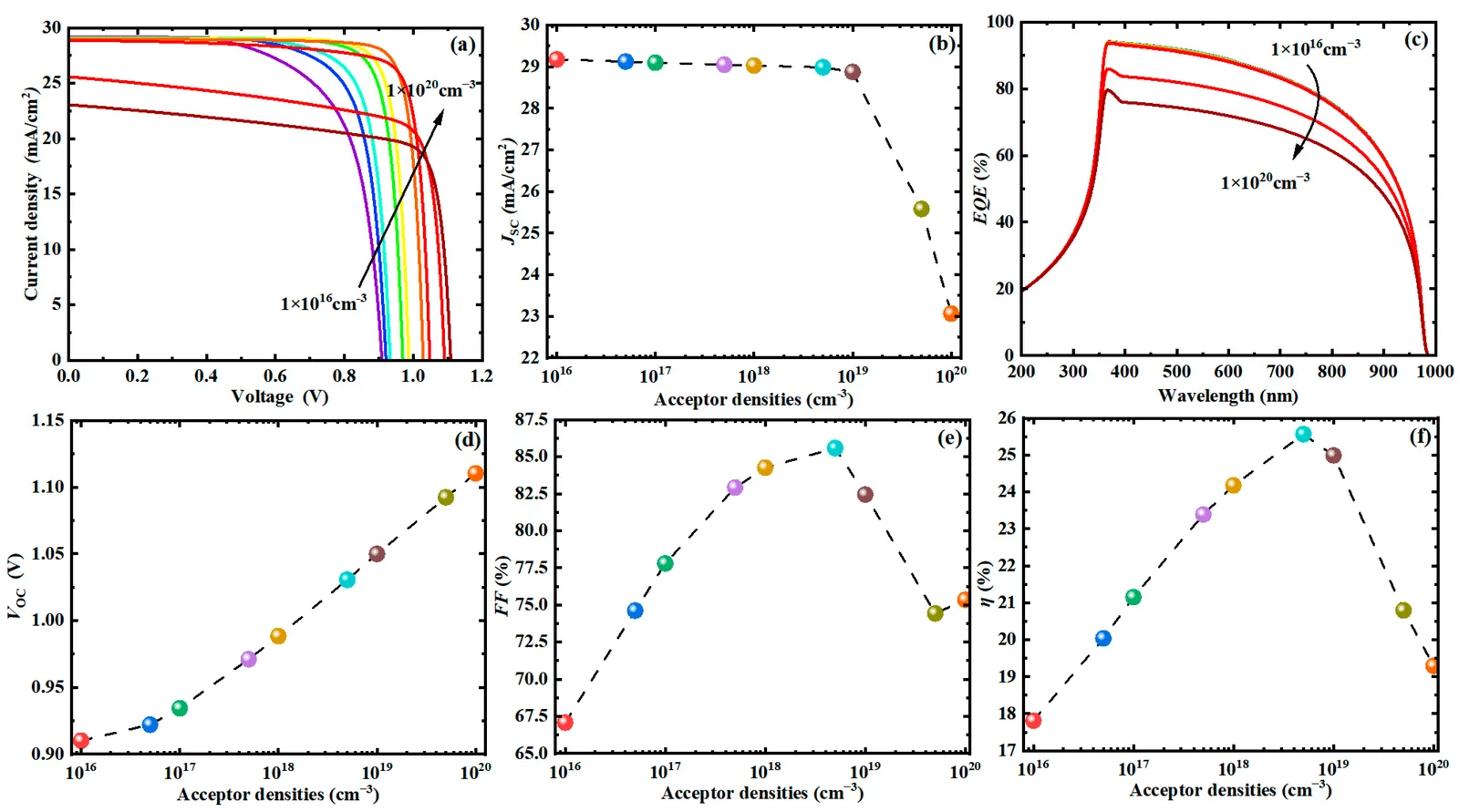
(**a**) JV curves of PSCs with different acceptor densities. The corresponding (**b**) *J*_SC_, (**c**) EQE, (**d**) *V*_OC_, (**e**) *FF*, and (**f**) *η* of solar cells. (**b**–**f**) The associated parameters: *J*_SC_, EQE, *V*_OC_, *FF*, and *η*.

**Figure 3:**
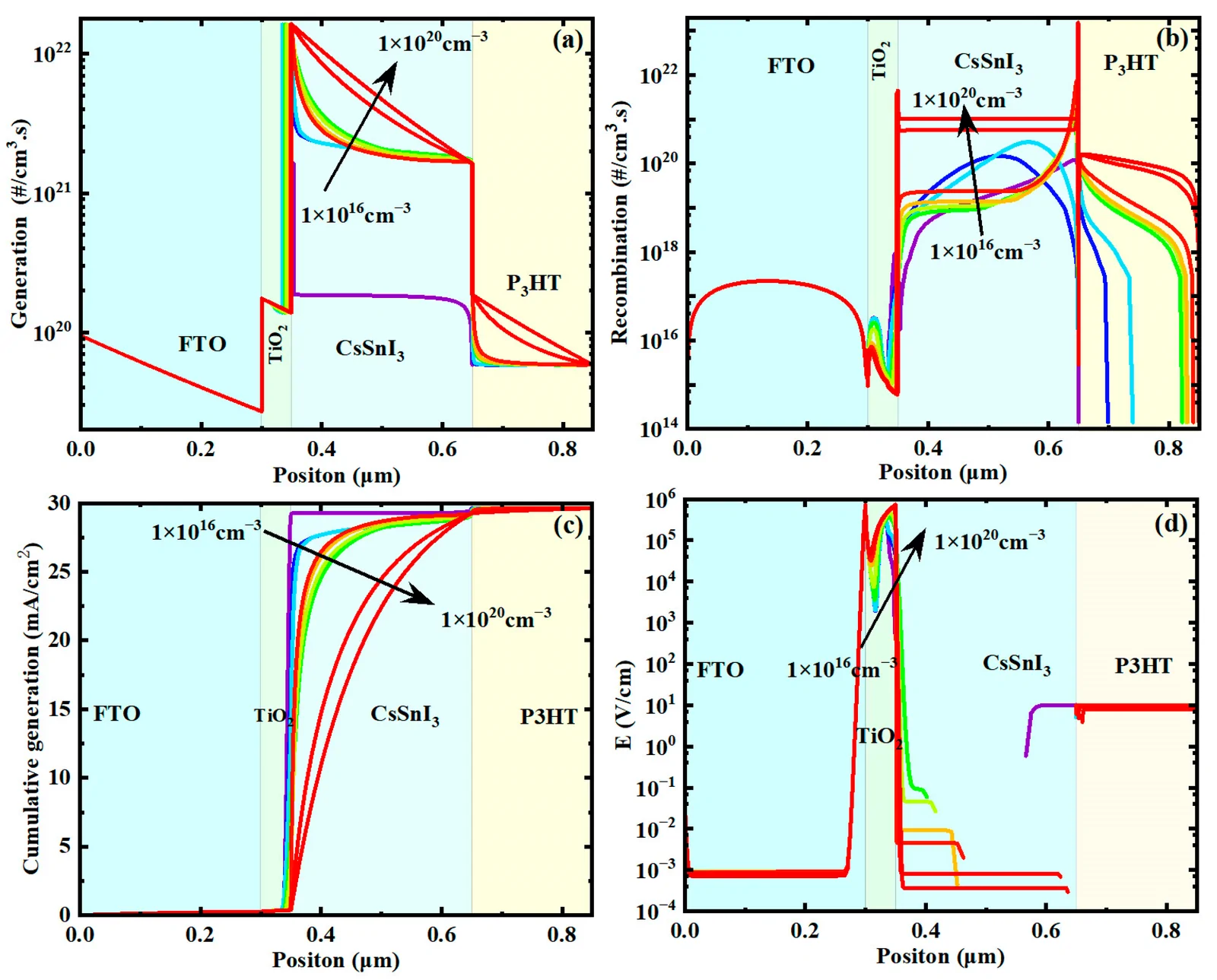
Spatial distribution of (**a**) carrier generation rate, (**b**) recombination rate, (**c**) cumulative generation and recombination, and (**d**) built-in electric field within the FTO/TiO_2_/CsSnI_3_/P3HT/Electrode device at different *N*_A_ in the CsSnI_3_ absorber layer.

To investigate the influence of the doping level within the perovskite absorber on device performance, we systematically varied the acceptor concentration (*N*_A_) of the CsSnI_3_ layer from 10^16^ cm^−3^ to 10^19^ cm^−3^. Figure 2 summarizes the corresponding photovoltaic response of the FTO/TiO_2_/CsSnI_3_/P3HT/Electrode devices. As depicted in Figure 2a, the current density–voltage (*J–V*) characteristics exhibit a pronounced evolution with increasing *N*_A_. At low doping concentrations, the *J*–*V* curve displays a relatively low open-circuit voltage (*V*_OC_) but a high short-circuit current density (*J*_SC_). As the acceptor density increases, the *V*_OC_ rises significantly, which is reflected in the rightward expansion of the *J–V* curve along the voltage axis. Concurrently, the curvature of the *J–V* curve near the maximum power point changes noticeably, indicating an alteration in the fill factor (*FF*). At excessively high *N*_A_ values (e.g., >10^19^ cm^−3^), the *J–V* curve shows a distinctive “saturation” or “roll-over” behavior at forward bias, suggesting that severe recombination or series resistance effects begin to dominate the charge transport process. This behavior is directly correlated with the spatial distribution of carrier generation and recombination illustrated in Figure 3a,b. At low *N*_A_, the generation rate is relatively uniform across the absorber bulk, while the recombination rate remains minimal, allowing efficient carrier collection. As *N*_A_ increases, the generation profile shifts toward the front interface due to enhanced absorption, but the recombination rate rises sharply, particularly near the P3HT interface, leading to the observed roll-over.

Figure 2b quantitatively illustrates the variation in *J*_SC_ with acceptor density. It is observed that *J*_SC_ maintains a relatively high level at low acceptor concentrations (below ~10^18^ cm^−3^). However, once *N*_A_ exceeds a critical threshold (approximately 10^19^ cm^−3^), *J*_SC_ begins to decrease monotonically. This degradation in current density can be rationalized by the enhanced Shockley–Read–Hall (SRH) recombination, as a higher doping concentration introduces more ionized impurities, which act as effective recombination centers and shorten the carrier diffusion length. Figure 3c further substantiates this interpretation by revealing the spatial evolution of the cumulative generation and recombination rates. At low *N*_A_ (10^16^ cm^−3^), the cumulative generation curve rises steeply and reaches a high plateau, while the cumulative recombination remains negligible, indicating that most photogenerated carriers are successfully collected. In contrast, at high *N*_A_ (10^19^ cm^−3^), the cumulative generation saturates at a lower value, and the cumulative recombination curve rises much earlier and more steeply, reflecting severe carrier loss before reaching the electrodes. The increasing overlap between the generation and recombination spatial profiles directly explains the *J*_SC_ deterioration with rising doping concentration.

To elucidate the underlying physical mechanism behind the *J*_SC_ decline, we examine the external quantum efficiency (EQE) spectra presented in Figure 2c. The EQE response directly reflects the spectral utilization efficiency of the device. At low acceptor concentrations, the EQE profile exhibits a broad and high plateau across the visible spectrum, indicative of efficient carrier generation and collection. As *N*_A_ increases, the EQE drops progressively, particularly in the long-wavelength region (near the bandgap edge of CsSnI_3_). This wavelength-dependent attenuation is a critical signature: photons with longer penetration depths are absorbed further away from the ETL (TiO_2_). In a highly doped absorber, the minority carrier (electron) diffusion length shrinks substantially due to increased trap-assisted recombination; consequently, photogenerated carriers in the bulk region recombine before reaching the TiO_2_/CsSnI_3_ interface. This reduction in carrier collection efficiency directly accounts for the decreased *J*_SC_.

Furthermore, Figure 2d presents the detailed simulated evolution of the *V*_OC_ as a function of *N*_A_ in the CsSnI_3_ absorber layer, with *N*_A_ ranging from 1.0 × 10^16^ cm^−3^ to 1.0 × 10^20^ cm^−3^. Over this entire range, *V*_OC_ monotonically increases from 0.910 V to 1.225 V, indicating that the built-in potential enhancement continuously governs the voltage behavior within the investigated doping interval, without any signs of saturation or roll-off. Quantitatively, the curve can be divided into three characteristic regions. In the low-doping regime (from 1.0 × 10^16^ cm^−3^ to approximately 1.0 × 10^17^ cm^−3^), *V*_OC_ rises from 0.910 V to 0.955 V, yielding an increment of 45 mV over one order-of-magnitude change in concentration. According to the classical p–n junction relation $\Delta V_{\mathrm{OC}}=\frac{kT}{q}\ln(N_{A2}/N_{A1})$, the theoretical gain at room temperature is about 60 mV per decade [11]. The slightly lower observed value (45 mV) suggests that space-charge region recombination still contributes to some extent at this doping level, partially offsetting the built-in potential gain. Figure 3d shows that at these low doping levels, the built-in electric field is relatively weak and distributed broadly across the absorber, resulting in moderate carrier drift velocities. The corresponding recombination rate remains low, which is consistent with the *V*_OC_ approaching but not fully reaching the ideal theoretical limit. In the intermediate concentration range (from 1.0 × 10^17^ cm^−3^ to 1.0 × 10^18^ cm^−3^), *V*_OC_ increases from 0.955 V to 1.045 V, giving a gain of 90 mV, which corresponds precisely to one decade of doping variation. This increment closely approaches the theoretical 60 mV/decade slope, indicating that the built-in potential enhancement is most efficient in this doping window and that SRH recombination has not yet become a significant limiting factor. Consistent with this, Figure 3d reveals that in this intermediate regime the built-in electric field reaches its optimal strength and spatial distribution (sufficiently strong to efficiently separate photogenerated carriers), yet not so intense as to induce significant trap-assisted recombination. The generation and recombination profiles remain well separated, ensuring effective carrier collection. Therefore, this region can be regarded as the optimal doping range for device operation, where the *V*_OC_ improvement aligns well with theoretical predictions. At high doping levels (from 1.0 × 10^18^ cm^−3^ to 1.0 × 10^18^ cm^−3^), *V*_OC_ continues to climb from 1.045 V to 1.225 V. The two-order-of-magnitude increase in concentration yields a total gain of 180 mV, corresponding to an average slope of 90 mV/decade, which exceeds the ideal 60 mV/decade. This super-linear growth deserves particular attention. Under extremely high doping, the depletion width becomes very narrow, which may enhance tunneling-assisted recombination and alter the dominant recombination mechanism, thereby causing the *V*_OC_ dependence on *N*_A_ to deviate from the classical p–n junction model. In summary, *V*_OC_ exhibits a strong positive correlation with $N_{A}$ across the entire simulated range, and the logarithmic dependence holds strictly in the 10^17^ to 10^18^ cm^−3^ interval. At extremely high acceptor concentrations, additional physical processes, such as quantum effects and tunneling recombination, may introduce extra voltage gains beyond classical expectations. These findings provide a quantitative basis for understanding and optimizing the voltage output of CsSnI_3_-based perovskite solar cells.

The fill factor (*FF*) in Figure 2e initially improves with increasing doping concentration, benefiting from an enhanced built-in electric field that facilitates efficient charge extraction and reduces carrier accumulation at the interfaces. However, as *N*_A_ continues to rise beyond the optimal range (~10^16^–10^17^ cm^−3^), the *FF* begins to deteriorate sharply. This decline is primarily attributed to the increased trap-assisted recombination and the consequent rise in the recombination current, which degrades the diode ideality factor and introduces additional voltage losses during device operation. Figure 3 corroborates this by showing that at high doping levels, the recombination rate increases dramatically across the entire absorber bulk, not just at the interfaces. The overlap between the generation and recombination spatial profiles becomes substantial, meaning that a large fraction of photogenerated carriers recombines before being extracted. This increased recombination current directly reduces the *FF*, as it lowers the voltage at which the maximum power point can be achieved.

Combining the trends of *J*_SC_, *V*_OC_, and *FF*, the overall power conversion efficiency (*η*) in Figure 2f exhibits a characteristic “bell-shaped” dependence on the acceptor density. The efficiency peaks at an optimal *N*_A_ of approximately 10^19^ cm^−3^, where a favorable balance between the improved built-in potential (enhancing *V*_OC_ and *FF*) and the acceptable recombination loss (maintaining reasonable *J*_SC_) is achieved. For *N*_A_ below this optimum, the insufficient built-in potential restricts *V*_OC_; for *N*_A_ exceeding this value, the dramatic loss in *J*_SC_ and *FF*, stemming from severe SRH recombination and reduced carrier lifetime, overrides the voltage gains, leading to a sharp efficiency drop. Therefore, a moderate acceptor doping level is essential to optimize the overall performance of CsSnI_3_-based perovskite solar cells.

**Figure 4:**
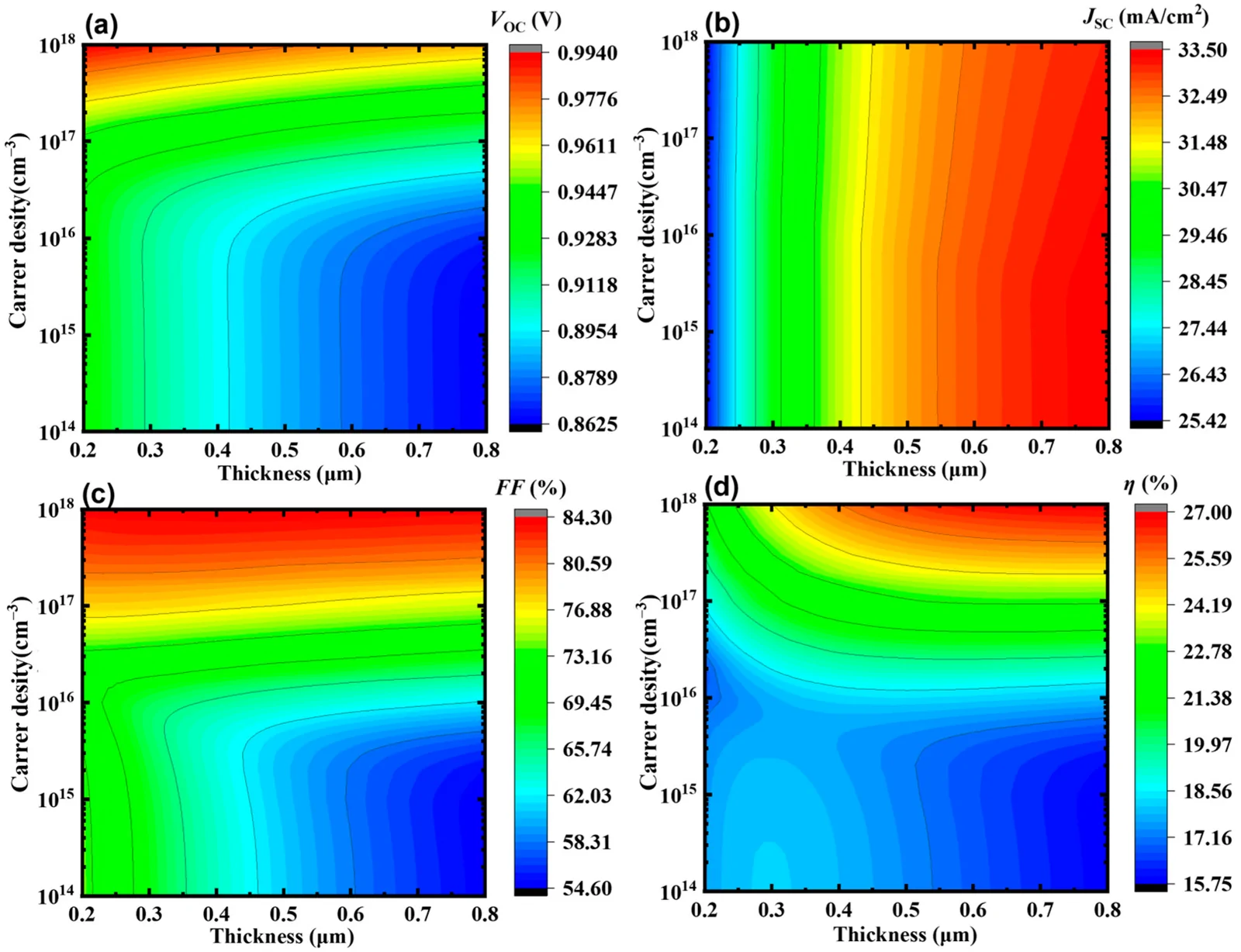
Impact of thickness and carrier concentration of the absorber layer on device performance of CsSnI_3_-based PSCs: (**a**) *V*_OC_, (**b**) *J*_SC_, (**c**) *FF*, (**d**) *η*.

The device performance of thin-film solar cells is critically dependent on both the absorber layer thickness and the carrier doping concentration [46]. In this study, we systematically evaluate the influence of the CsSnI_3_ absorber layer thickness (ranging from 0.2 to 0.8 µm) and acceptor doping concentration (*N*_A_, varying from 10^14^ to 10^18^ cm^−3^) on the four key photovoltaic parameters: *V*_OC_, *J*_SC_, *FF*, and *η*. The results presented in Figure 4 demonstrate that *V*_OC_ increases monotonically with rising doping concentration across the entire investigated range, a behavior attributed to the enhanced built-in electric field resulting from increased band bending at the p–n junction. In contrast, *V*_OC_ exhibits a slight downward trend with increasing absorber thickness, which can be ascribed to the intensified non-radiative recombination in thicker layers, where photogenerated carriers must travel longer distances before collection.

In contrast to the behavior of *V*_OC_, the *J*_SC_ is predominantly governed by the absorber layer thickness. As the thickness increases, the optical path length extends, leading to enhanced photon absorption and consequently a significant improvement in *J*_SC_. This trend reflects the improved light-harvesting capability of thicker absorber layers, which generate a larger number of photogenerated carriers. However, the doping concentration exerts a relatively minor direct influence on *J*_SC_, a characteristic typical of materials where optical generation dominates over transport limitations. Only at excessively high doping levels (exceeding 10^19^ cm^−3^) does a noticeable degradation occur, as severe SRH recombination, induced by the abundant ionized impurities, shortens the minority carrier diffusion length and impairs the carrier collection efficiency, as previously illustrated in Figure 2b.

The *FF* achieves its optimal values under moderately high doping concentrations and relatively thin absorber layers. In this regime, the strengthened internal electric field facilitates efficient charge extraction and minimizes carrier accumulation at the interfaces. However, when the absorber layer becomes excessively thick or the doping level falls too low, the *FF* deteriorates markedly. The degradation at excessive thickness arises from increased series resistance and enhanced bulk recombination, both of which reduce the voltage at which the maximum power point is achieved. At insufficient doping levels, the weak built-in field fails to provide adequate driving force for charge separation, leading to increased recombination losses and a consequent decline in *FF*.

Combining the individual trends of *V*_OC_, *J*_SC_, and *FF*, the *η* exhibits a bell-shaped distribution, with the peak efficiency emerging at an optimal balance between thickness and doping concentration. Within this optimized window, the device simultaneously benefits from sufficient light absorption (ensuring a high *J*_SC_), a strong built-in potential (providing a high *V*_OC_), and moderate recombination losses (maintaining a good *FF*). Conversely, deviations from this optimum, whether through insufficient thickness or doping (limiting photon capture and electric field strength) or excessive values (inducing severe recombination and transport losses), result in a sharp decline in overall device performance. These findings underscore the critical importance of co-optimizing both absorber thickness and doping concentration to achieve high-efficiency CsSnI_3_-based perovskite solar cells.

**Figure 5:**
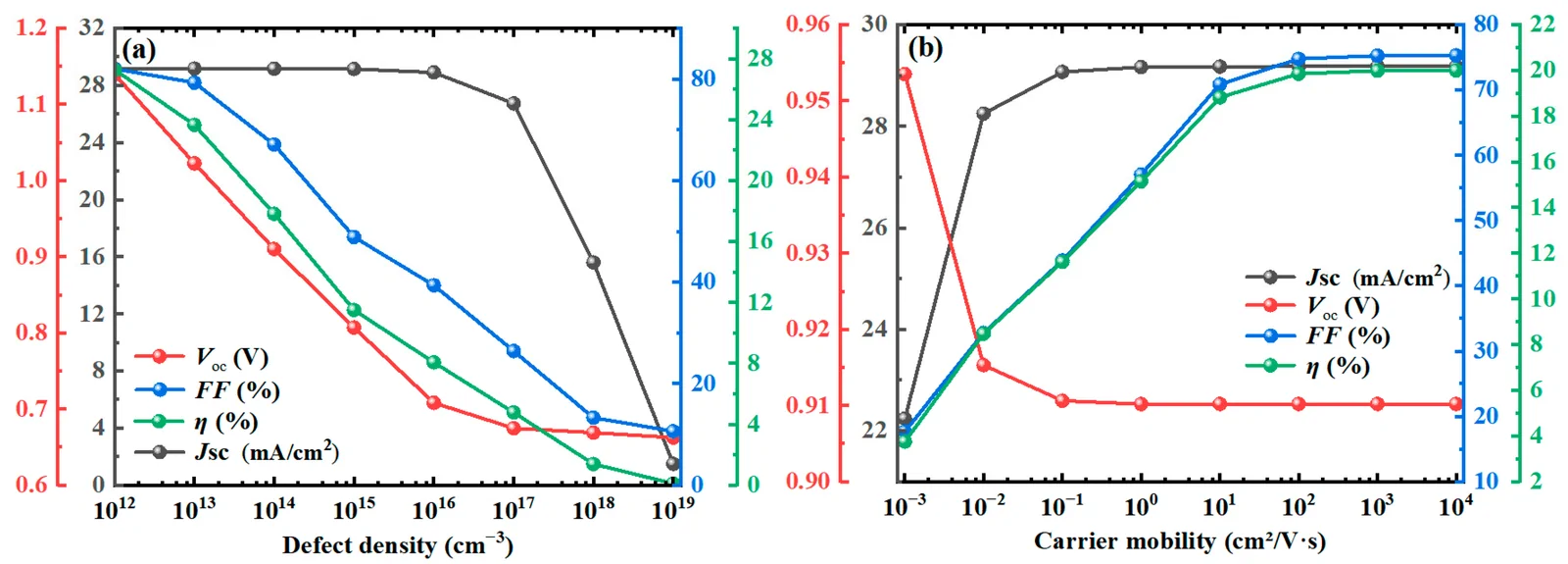
Effect of (**a**) defect density and (**b**) carrier mobility of CsSnI_3_ absorber layer on device performance.

Defect density (*N*_t_) is a critical parameter governing the performance of CsSnI_3_-based PSCs. Figure 5a systematically presents the evolution of the four key photovoltaic parameters, *V*_OC_, *FF*, *J*_SC_ and *η*, as the absorber defect density increases from 10^12^ to 10^19^ cm^−3^. With rising defect density, *V*_OC_, *FF*, and *η* exhibit a continuous degradation. In the low-defect-density regime (10^12^–10^14^ cm^−3^), the decline is relatively moderate; however, once *N*_t_ exceeds 10^14^ cm^−3^, the degradation rate accelerates markedly, indicating that non-radiative recombination losses begin to dominate. Notably, *J*_SC_ remains constant at 28.0 mA/cm^2^ across the 10^12^–10^16^ cm^−3^ range, suggesting that photogenerated carrier generation is primarily governed by the optical absorption properties of the absorber layer, and that recombination-induced carrier losses at low defect densities are not yet sufficient to compromise the collection efficiency. Beyond the critical threshold of 10^16^ cm^−3^, however, *J*_SC_ drops abruptly from 28.0 mA/cm^2^ to nearly zero, while *V*_OC_ continues to decline, *FF* falls from 40% to approaching zero, and *η* plummets from 8% to virtually nil. The underlying physical mechanism resides in the excessive defect density, which introduces a large number of non-radiative recombination centers and substantially exacerbates SRH recombination. This not only increases the space-charge region recombination current (thereby reducing *V*_OC_), but also drastically shortens the minority carrier diffusion length, causing photogenerated carriers to recombine before reaching the electrodes (thus impairing both *J*_SC_ and *FF*). In summary, to achieve high-performance CsSnI_3_ solar cells, the absorber defect density must be strictly maintained below 10^16^ cm^−3^ to suppress the comprehensive performance losses induced by SRH recombination.

Carrier mobility (*μ*) is a critical parameter governing the charge transport efficiency of CsSnI_3_-based perovskite solar cells. Figure 5b systematically presents the evolution of the four key photovoltaic parameters, *V*_OC_, *FF*, *J*_SC_ and *η*, as the absorber carrier mobility increases from 10^−3^ to 10^4^ cm^2^/V·s. With increasing mobility, *V*_OC_ exhibits an initial decline followed by a stabilization trend: at mobility values below 10^−1^ cm^2^/V·s, the sluggish carrier transport leads to carrier accumulation at the interfaces, exacerbating interfacial recombination and thereby reducing *V*_OC_; once the mobility exceeds 10^−1^ cm^2^/V·s, carrier transport becomes sufficiently rapid to suppress interfacial recombination, and *V*_OC_ stabilizes at approximately 0.91 V, indicating that a moderate mobility is adequate to maintain a high quasi-Fermi level splitting, with further mobility enhancement yielding only marginal gains. In stark contrast, *FF*, *J*_SC_ and *η* all exhibit continuous upward trends with increasing mobility: when the mobility exceeds 10^−1^ cm^2^/V·s, *J*_SC_ can be maintained at approximately 29 mA/cm^2^, attributable to the fact that higher mobility ensures rapid transport of photogenerated carriers to their respective electrodes, substantially reducing bulk recombination losses and thereby enabling highly efficient carrier collection; *FF* rises steadily from 22.0% at 10^−3^ cm^2^/V·s to 75.0% at 10^4^ cm^2^/V·s, while *η* increases significantly from 4.0% to 21.0%, with these improvements being particularly pronounced in the low-mobility regime (10^−3^ to 10 cm^2^/V·s), beyond which the gains gradually diminish. The underlying physical mechanism is that at low mobility, sluggish carrier transport causes photogenerated carriers to recombine before reaching the electrodes, while simultaneously increasing the series resistance, thereby severely limiting *FF*, *J*_SC_ and *η*; as mobility improves, carrier transport becomes more efficient, recombination losses are reduced, and series resistance decreases, resulting in sustained performance enhancement; however, once the mobility exceeds approximately 1 cm^2^/V·s, transport is no longer the primary performance-limiting factor, and further mobility improvements yield only marginal benefits. In summary, to ensure satisfactory device performance in CsSnI_3_ solar cells, the absorber carrier mobility should be maintained at least above 1 cm^2^/V·s to guarantee efficient carrier collection and minimize transport losses.

**Figure 6:**
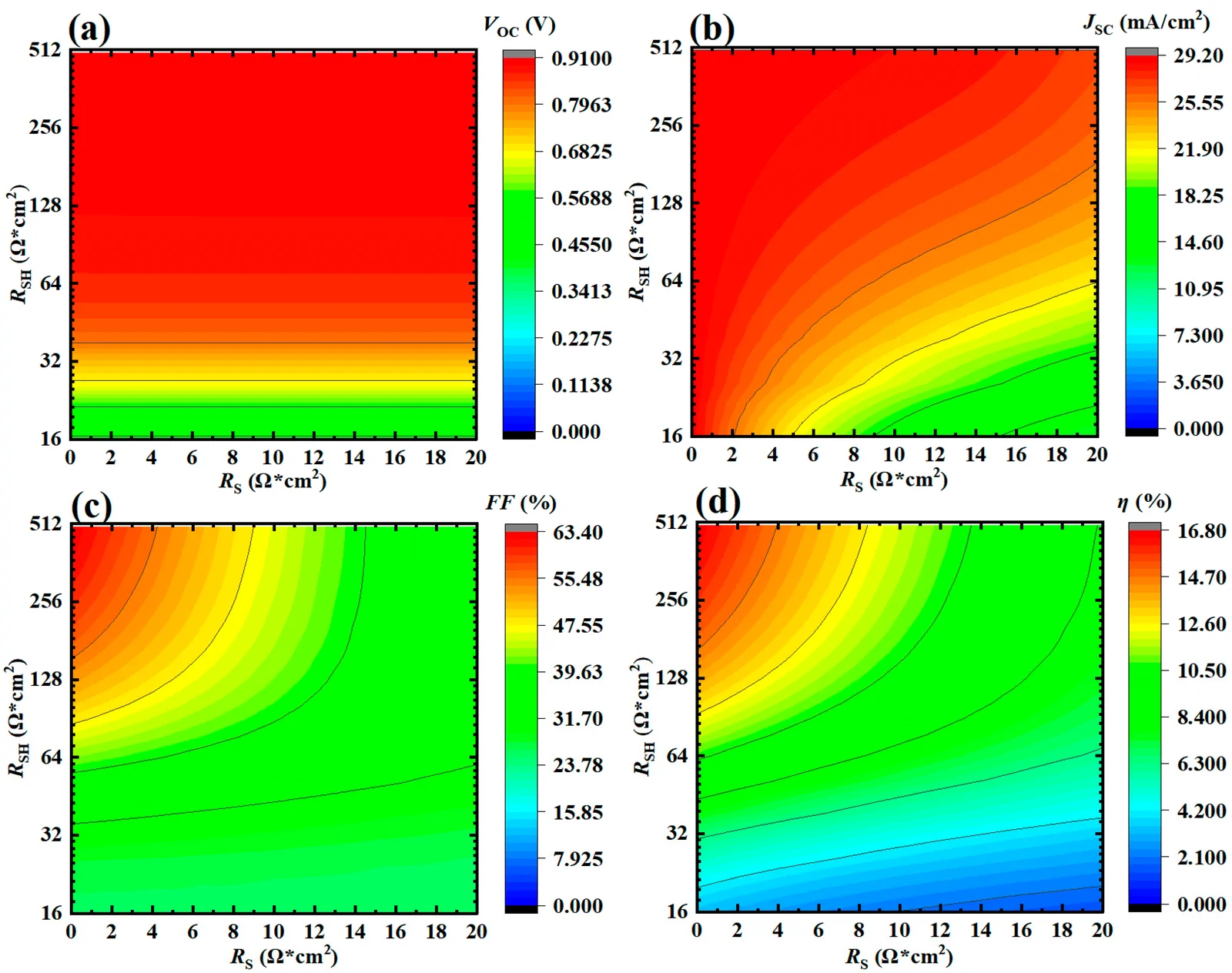
Contour-style plots illustrating the concurrent influence of changes in series resistance and shunt resistance on photovoltaic performance metrics: (**a**) *V*_OC_, (**b**) *J*_SC_, (**c**) *FF*, (**d**) *η*.

The equivalent circuit parameters of thin-film solar cells, series resistance (*R*_S_) and shunt resistance (*R*_SH_), play a critical role in determining device performance. In this study, we systematically evaluate the influence of *R*_S_ (ranging from 0 to 0.20 Ω·cm^2^) and *R*_SH_ (ranging from 16 to 500 Ω·cm^2^) on the four key photovoltaic parameters (*V*_OC_, *FF*, *J*_SC_ and *η*) of CsSnI_3_ solar cells. As shown in Figure 6, *V*_OC_ remains nearly unaffected across the entire *R*_S_ range. This is because, under open-circuit conditions, no current flows through the series resistance, and thus no voltage drop occurs across it; consequently, *V*_OC_ is intrinsically governed by the band structure and interfacial recombination characteristics of the absorber. *J*_SC_ is also influenced by *R*_S_ to some extent, though the effect is relatively modest, under short-circuit conditions, the voltage drop across the series resistance exists but its attenuation of the total current is limited; only at extremely high *R*_S_ values do resistive losses begin to compromise the current output.

In stark contrast, both *FF* and *η* exhibit extreme sensitivity to *R*_S_. As *R*_S_ increases from 0 to 0.20 Ω·cm^2^, *FF* drops sharply from approximately 63.4% to near zero, accompanied by a catastrophic decline in η from about 16.8% to virtually nil. This degradation arises from the significant voltage drop induced by series resistance during device operation, which compresses the voltage window at the maximum power point and severely undermines the output capability. When *R*_S_ is maintained below 2 Ω·cm^2^, FF remains above 60% and *η* stays at a relatively high level; however, once *R*_S_ exceeds 8 Ω·cm^2^, resistive losses become the dominant limiting factor, leading to abrupt performance deterioration. Regarding *R*_SH_, its influence is mainly manifested at relatively low values (≤128 Ω·cm^2^). A low shunt resistance provides an additional leakage path for photogenerated current, resulting in measurable declines in both *V*_OC_ and *FF*. When *R*_SH_ is sufficiently large (>128 Ω·cm^2^), its impact on device performance becomes essentially negligible.

In summary, to achieve high-efficiency CsSnI_3_ solar cells, the *R*_S_ should be strictly controlled below 2 Ω·cm^2^, and the shunt resistance maintained above 128 Ω·cm^2^, so as to minimize the adverse effects of resistive and leakage losses on fill factor and efficiency.

## 4. Conclusions

In this work, we have performed a systematic numerical simulation of CsSnI_3_-based perovskite solar cells using SCAPS-1D, with particular attention to the coupled effects of absorber doping concentration, thickness, defect density, carrier mobility, and parasitic resistances on device performance. The simulation results reveal that the acceptor doping concentration exhibits a clear dual influence: moderate doping enhances the built-in electric field and thus improves the *V*_OC_, but once the concentration exceeds approximately 10^18^ cm^−3^, excessive doping significantly intensifies Shockley-Read-Hall recombination, leading to a sharp decline in both *J*_SC_ and *FF*. Consequently, the optimal doping level is found to be around 10^19^ cm^−3^, where the best trade-off among the key parameters is achieved. Absorber thickness directly governs light harvesting; increasing thickness boosts the *J*_SC_, yet it also aggravates bulk recombination and *R*_S_, and overly thick films ultimately impair device performance. In practice, a thickness in the range of 0.3–0.5 μm appears reasonable, with the exact value requiring co-optimization with the doping concentration. Defect density constitutes another critical limiting factor and must be strictly kept below 10^16^ cm^−3^; beyond this threshold, non-radiative recombination becomes dominant and all performance metrics deteriorate rapidly. Carrier mobility, on the other hand, needs to exceed 1 cm^2^/V·s to ensure efficient transport and collection, while further increases in mobility yield diminishing returns. Moreover, series and shunt resistances exert notable influences on fill factor and efficiency; we recommend keeping series resistance below 2 Ω·cm^2^ and shunt resistance above 128 Ω·cm^2^ to minimize resistive and leakage losses. Overall, achieving high-efficiency CsSnI_3_ solar cells demands simultaneous consideration of doping, thickness, defect control, mobility enhancement, and contact quality. These parameters are not independent but rather interdependent, and only through synergistic optimization can the full potential of tin-based perovskite photovoltaic technology be fully realized.

## Data Availability

Data will be made available upon request.
